# Can practitioners use patient reported measures to enhance person centred coordinated care in practice? A qualitative study

**DOI:** 10.1186/s12955-018-1045-1

**Published:** 2018-12-04

**Authors:** Hannah Wheat, Jane Horrell, Jose M. Valderas, James Close, Ben Fosh, Helen Lloyd

**Affiliations:** 1 Department of Sociology, Philosophy and Anthropology, University of Exeter, Exeter, UK; 2 Community and Primary Care research group, Plymouth University Peninsula Schools of Medicine & Dentistry, Plymouth, UK; 3 University of Exeter Medical School, Exeter, UK; 4 School of Psychology, University of Plymouth, Room A206 Portland Square, Plymouth, PL6 8BX UK

**Keywords:** Person centred coordinated care, Patient reported measures, Practitioner, Communication, goals, decision-making, Care planning, transitions, clinical practice

## Abstract

**Background:**

To ascertain whether person centred coordinated care (P3C) is being delivered in healthcare services, components relating to the construct need to be measured. Patient reported measures (PRMs) can be used to provide a measurement of patients’ experiences of P3C. Traditionally, they have been used to assess whether interventions are delivering P3C. Recently there has been an increased interest in using them to directly enhance P3C in clinical practice by, for example, improving practitioner-patient communication. However, there is limited research available on how P3C can be implemented in practice. This study aimed to extend this literature base by exploring how professionals use PRMs to enhance P3C.

**Methods:**

Cross sectional thematic analysis of 26 semi-structured interviews with a variety of professionals who have experience of how PRMs can be used to make improvements to P3C. Inductive themes were mapped onto components of P3C care that fell under five established domains of P3C (Information and Communication; My Goals/Outcomes; Decision making; Care Planning and Transitions) to explore whether and how individual components of P3C were being improved through PRMs. Barriers and facilitators that affected the delivery and the results of the PRMs were also identified.

**Results:**

Three P3C domains (Information and Communication, My Goals/Outcomes and Care Planning) were mapped frequently onto themes generated by the participants’ interviews about PRM use. However, the domain ‘Decision Making’ was only mapped onto one theme and ‘Transitions’ was not mapped at all.

Participant reports suggested that PRM use by practitioners enhanced patients’ ability to self-manage, communicate, engage and reflect during consultations. Barriers to PRM use were related to a lack of a whole service approach to implementation.

**Conclusions:**

Practitioners use ***both*** PROMs and PREMs in various ways to improve different aspects of patient care. By sharing experiences professionals can benefit from each other’s learning and work together to extend the potential value that PRMs can offer to P3C delivery.

## Background

### Person Centred coordinated care

**Fig. 1:**
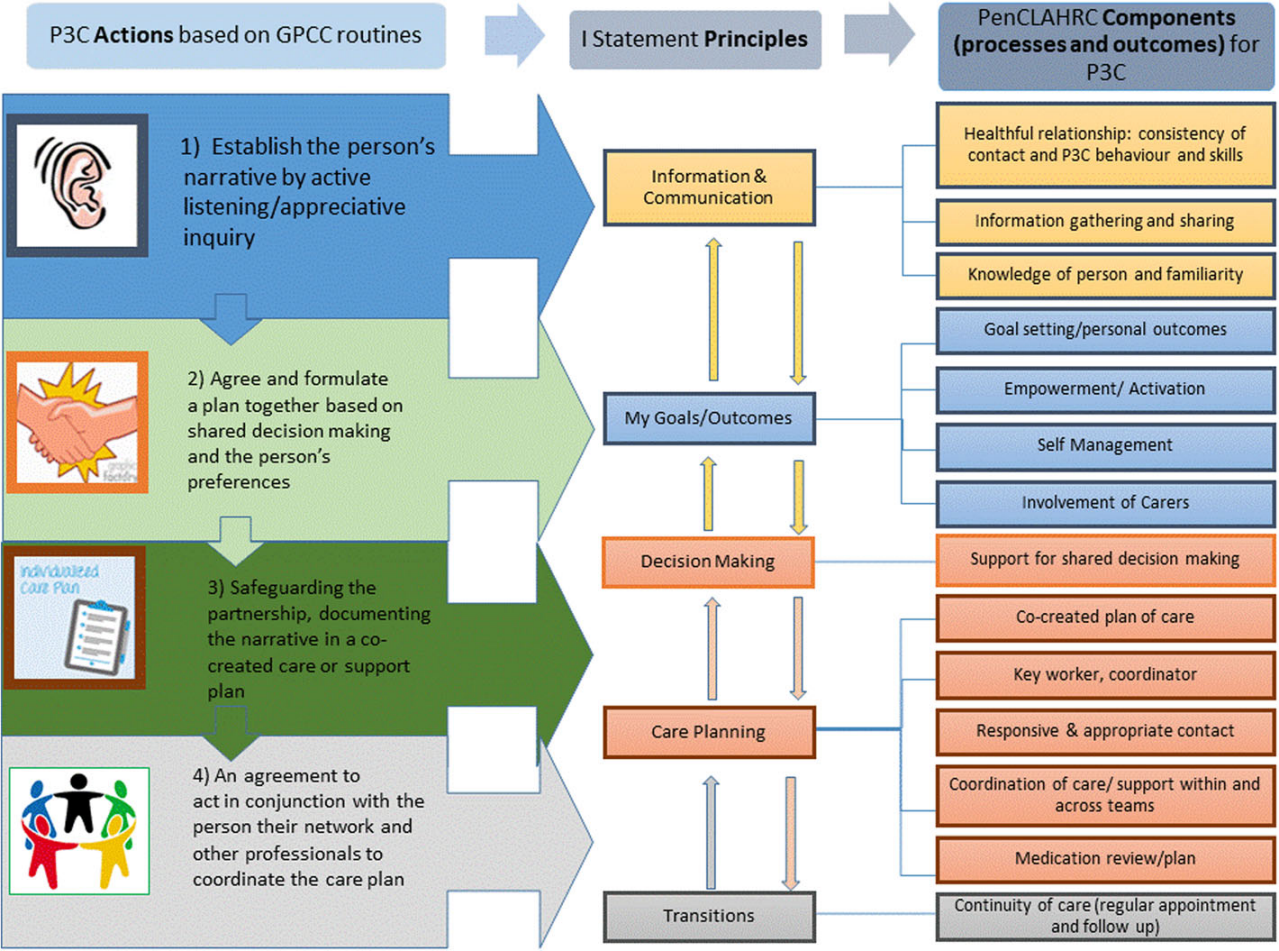
A model of P3C: translating P3C principles into action [4]

Person Centred Care (PCC) has been shown to improve processes within, and outcomes of, health care services and is a promising strategy for alleviating the current burdens imposed on health care services [1]. A specific model of Person Centred (Coordinated) Care (P3C) [2–5] has been developed and informed by service user “I” statements: narrative accounts of what they perceive to be good PCC [6], the House of Care Model [7] and research literature [5]. It is built upon five domains that are core to P3C: Communication and Information, (service user) Goals/Outcomes, Decision making, Care Planning and Transitions [2–5]. Health and social care professionals can apply these domains to practice by “working collaboratively with people who use services **(communication**); [by supporting] people to develop the knowledge **(information),** skills and the confidence they need to more effectively manage and make informed decisions **(decision making)** about their own health and health care; [by making care] coordinated **(transitions)** and tailored to their needs **(goals/outcomes**) and by ensuring that people are treated with dignity, compassion and respect **(communication)** through improved **care planning** and care delivery” ([8], p. 3 - domains added by authors). Within the aforementioned P3C model, each domain is broken down into its component parts and translated into four actions that can be performed in practice (see Fig. 1 below; the first three actions were developed by Ekman, Swedberg and Taft et al [9] from the Gothenburg centre for PCC. The fourth action, 'care coordination', was later added by Lloyd et al [3, 4]). One component of the domain ‘Information/Communication’, for example, is ‘knowledge of person and familiarity’. This component can be translated into action by a practitioner encouraging and actively listening to a patient’s own narrative account of their holistic health issues and care needs.

### Measuring person Centred coordinated care

Patient experience, most frequently measured using patient reported measures (PRMS), is arguably one of the most important ways for assessing whether P3C is being delivered [10]. Such measures probe P3C as either a general construct (e.g. the Person-Centred Coordinated Care Experience Questionnaire – P3CEQ) [11] or a sub-component of it, e.g. shared decision-making (e.g. through the OPTION observation tool [12]. PRMs can be used to elicit patient scores (and free text in some cases) of their experiences and outcomes (e.g. symptom states and behaviour) using numerical scales. Several variants fall under the umbrella term PRM: Patient Reported Experience Measures (PREMs) and Patient Reported Outcome Measures (PROMs), including also Individualised Patient Reported measures (iPROs), which are a particular type of PROMs. All of these are defined in existing literature [4, 13] and in an online compendium of PRMs that relate to P3C [5].

### How PRMs are used to inform healthcare

Although PRMs have been used predominantly in research to assess whether interventions have achieved intended outcomes [4, 14, 15] for patients and service delivery (including P3C), there is some evidence that they can serve as a mechanism for enhancing P3C directly. Specifically, PRM use has been shown in various health settings (including neurosurgery, oncology routine and palliative care to: enhance communication and the identification of patient problems; inform diagnoses; facilitate clinical decision-making and the monitoring of progress and to help empower patients to self-manage their care (e.g. [16–31]). In addition, the potential benefits of using PRMs for other domains of care have been explored and identified through the development of a conceptual framework describing the potential effects of using PROMs in chronic care management [32]. Within the framework, it is argued that ‘patient activation,’ for example, could be enhanced through PRM use by enabling practitioners to gauge current levels of activation and to understand *how* to improve upon those levels. For example, if the individual has low levels of confidence about self-managing their health care issues, the practitioner could work with the patient on improving this specific aspect of activation [32].

### Rationale for this study

There is increasing clarity over what P3C is and how it can be translated into practice [4]. This is important as P3C has been shown to improve health outcomes as well as processes and experiences of care [1]. There is also growing evidence that PRMs can provide a means of directly enhancing the delivery of P3C in healthcare settings, as well as measure it. However, there are many challenges limiting PRM application in clinical practice [21, 26]. Consequently, there are potentially huge benefits from better understanding how PRMs are being utilised in different contexts; whether this is part of a research project, driven by service improvement activities or part of routine clinical practice, so that services can learn from one another and levels of P3C can be improved upon. However, practitioners are increasingly using PRMs to improve their delivery of healthcare in isolation and without clear guidance [15, 27, 30, 31], whereas clinical commissioning initiatives and NHS programmes of care for the routine use of PRMs are often not shared nor widely disseminated [4]. This study sought to elicit professionals’ experience-based perspectives on whether PRMs can enhance P3C in practice and what factors impede or facilitate this process, so that services can benefit from each other’s experiences and the potential (and limitations) of PRMs to improve P3C can be further understood, addressed and realised in the future. This study builds upon, but differs from existing research on this topic area as: (1) while existing studies have focused on PRM use in a particular health setting, we sought perspectives from a range of practitioners and non-practitioners, from a number of different institutional settings, to elicit a broader understanding of how PRMs can be used to improve P3C in practice, (2) the study aimed to look at the use of different types of PRMs (PROM, PREM and iPROs), rather than one type of PRM and (3) we intended to capture real life instances of how PRMs could improve the delivery of **each** P3C domain, rather than focusing on the actualisation of individual components or building a theoretical model of what PRMs could offer. This would enable areas of strength or potential gaps in PRMs ability to enhance P3C in practice to be identified.

## Methods

### Study design

A mix of primary and secondary qualitative data, including a total of 26 semi-structured interviews were collected to identify 1) how practitioners are using PRMs to enhance their practice and 2) barriers and facilitators that impacted on these applications.

### Data collection

#### Primary data collection

Fourteen participants were purposively selected for this study to ensure a range of experiences was captured. Participants had either a single role, or a combination of roles as a researcher, commissioner or practitioner, programme or network manager or lead, or local government director. The interviewee was still viewed as a practitioner even if they were not currently employed in that role. The type of health issues they were primarily concerned with varied, with some participants focusing on the general health of people within a population, their locality or institution and others trying to improve care for specific conditions/stages of life, such as cancer and care for people reaching the end of life. Potential participants were identified by reviewing media coverage of PRM use, grey literature and through suggestions made by interviewees and fellow researchers. Consequently, the type of professional roles and institutional settings sampled in the study was not limited by an over reliance on one researcher’s initial points of interest or knowledge of the subject area or a particular heath setting. The only inclusion criteria were that the potential participant had been involved in the design, implementation, or study of at least one PRM in the last year with the aim of improving healthcare delivery. Potential participants were initially contacted by email by author 1 and sent an information sheet and consent form to review. Participants, who had given informed consent, were interviewed by Authors 1 and 2 between February and July 2016.

#### Secondary data collection

During initial reviews of the primary data, an emerging analytic interest became practitioners’ accounts of how PRMs had been implemented in practice (by themselves or another practitioner) to improve P3C. While the primary dataset provided rich accounts of such experiences from practitioners (*n* = 6) and non-practitioners (*n* = 8), the authors, after an initial analysis of the data, felt the study would benefit from the dataset being extended through more first-hand practitioner accounts. Consequently, we conducted a secondary analysis of an existing dataset, which included interviews with 14 healthcare practitioners who were currently practicing, conducted between February 2016 and April 2017. These practitioners were implementing new models of care in the South West at the time of their interview. We used the same methodological approach for this dataset that we had used for the primary dataset. The participants were GPs, community workers, nurses and care-planning professionals with medical and non-medical backgrounds. The practitioners were working within the primary care sector and consequently, dealt with a range of physical and mental health issues and often had to coordinate care with secondary and third sector services. The new model of care they were implementing targeted long-term conditions. Consequently, a large number of the patients that they saw were elderly with complex needs; requiring a complete P3C approach.

These participants’ interviews were screened for reference to PRM use. 12 out of the 14 interviews contained at least one reference to the use of PRMs.

#### Cumulative data set

Twenty-six interviews with various professionals working in, or with, healthcare services. 18 of the 26 professionals were clinicians (and may or may not have had additional roles). Table 1 provides an overview of the sample’s characteristics. The cumulative dataset included data from 10 males and 16 females; 18 professionals who were practitioners and 8 who were not and 24 British and 2 non-British participants. The diversity of institutional settings, roles and health interests within our sample provided a strong opportunity for all aspects of P3C to be mapped.

**Table 1:** Sample characteristics

	Practitioner/Non-practitioner	Female/Male	Location
Primary data set	Practitioner = 6	Males *n* = 6	UK *n* = 12
	Non- practitioner *n* = 8	Female *n* = 8	Other *n* = 2
Total participants	*n* = 14		
Secondary data set	Practitioner *n* = 12	Males *n* = 3	UK *n* = 12
	Non-practitioner *n* = 0	Female *n* = 9	Other *n* = 0
Total participants	*n* = 12		
Cumulative data set	Practitioner *n* = 18	Males *n* = 10	UK *n* = 24
	Non-practitioner *n* = 8	Female *n* = 16	Other *n* = 2
Total participants	*n* = 26		

#### Interview methods

All interviews lasted 30–60 min, were conducted using semi-structured topic guides and were audio recorded and transcribed. All primary data interviews were conducted over the telephone, whereas secondary data interviews were conducted as either face-to face or telephone interviews.

#### Analysis

To build a collective understanding of how PRMs were being used to enhance individual domains of P3C from our interview data, which derived from a diverse range of settings and professional roles, we used thematic analysis [33]. A method that enables patterns (themes) within interview data, which are relevant to a particular phenomenon or research question, to be identified and examined.

All interviews were transcribed, anonymised and assigned a unique identifier before being entered into NVivo for analysis. Author 1 began the analysis by first familiarising herself with the data. The primary data was then coded inductively and modified in NVivo until a set of codes, which exhaustively and accurately captured various forms of PRM use was created. This codebook informed the analysis of the secondary data. New inductive codes from the secondary dataset were added to the codebook and checked for in the primary dataset. Coded extracts across the entire data set were reviewed and compared so that codes that fitted together and provided a unique insight into PRM use to enhance P3C could be collated to form a theme. These themes were then reviewed in terms of whether the data supported the theme, whether the themes could be split into further sub-themes or whether themes could be collapsed. This process resulting in a robust pattern of themes and saturation point being reached (we could find no additional data to further develop our existing themes). Using the aforementioned model of P3C as a guide [2–5], themes were then mapped to P3C domains components [2–5] that relate to each of the five main domain headings, if they related to the fulfilment of that P3C activity. As every instance of a PRM being used to enhance a P3C domain was used in the thematic analysis and ‘counted’ to create a prevalence figure for each domain (see Table 2), irregular themes (in terms of how frequently they were reported) were also examined and are reported on later in the results section e.g. shared decision making (*n* = 1). A framework analysis approach was not adopted at the start, so that the themes were data driven, rather than influenced by a priori P3C themes. However, through the mapping process the P3C domains provided an objective ‘framework’ by which to organise themes and re-assess whether they best represented the data. Inter-rater reliability was checked by Author 2 performing a blind coding of 10% of the transcripts, coding differences were discussed during a meeting before the mapped P3C themes were finalised. Finally, barriers and facilitators for the uptake of PRMs in clinical practice were summarized.

**Table 2:** Coverage of P3C domains and components within the dataset

P3C Domains X is used to highlight P3C components that themes were not mapped onto (were not reported as being enhanced through PRM use).							
	Information and communication (Dataset: primary and secondary) *n* = 12		My goals/outcomes (Dataset: primary and secondary) *n* = 10	Decision-making (Dataset: primary) *n* = 1	Care planning (Dataset: primary) *n* = 10	Transitions *n* = 0	
Components of P3C		Consistency of contact, P3C behaviours and skills	Goal setting/outcomes	Support for Shared decision making	Co-created plan of care	X	Continuity of care (regular appointment and follow up)
		Information gathering and sharing	Empowerment/Activation		Key worker, coordinator		
		Knowledge of person and familiarity	Self-management		Responsive and appropriate contact		
	X	Involvement of carers			Coordination of care/support within and across teams		
					Medication (in this case treatment) review/plan		

## Results

The primary data generated themes for 4 out of the 5 P3C domains, with ‘Transitions’ not receiving any coverage. The secondary data yielded themes for 2 of the domains already mapped onto through the primary data (Information and Communication and Goals/outcomes). 3 of the 4 mapped P3C domains (Information and Communication, My Goals/Outcomes and Care Planning) were mapped frequently onto themes generated by the participants’ interviews about PRM use. However, the domain ‘Decision Making’ was only mapped onto one theme. Both datasets provided information on what factors served as a facilitator or barrier to practitioners’ applications of the PRMs.

In Table 2, a **X** has been assigned to P3C components [2–5] that themes were not mapped onto (were not reported as being enhanced through PRM use). If no **X** is assigned, the component was mapped onto (was enhanced through PRM use). A numerical figure for how many participants contributed to themes mapped to each domain is provided in the horizontal column headings. This figure indicates how prevalent the P3C domain was in the particpants accounts or, in other words, how many of the particpants reported a similar experience of PRM use. Detailed accounts of themes are provided in indivudalised sections below. In each section, the sequential ordering of the themes is based on their prevalence within the data set, with the most frequently presented themes provided first. Table 3 provides quotes to accompany the themes for the three domains that received most coverage.

**Table 3:** Quotes to accompany themes regarding PRM use

P3C Domains		
Information and communication	My goals/outcomes	Care planning
1a) *Facilitating the presentation of symptom change and current status* “Some people find it really helpful to do the [PAM] and the [WEMWBS] because it gives them a tool for focusing their questions and how they’re feeling”.	2a) *Self-management: PRMs can help practitioners identify issues and areas for improvement* “[The PAM] highlights areas they may think they are confident about, but in reality they’re not. If completed with conversation [you can] set goals to improve”.	*4a) Supporting health practitioners to provide tailored care* “They have identified through this huge [PROM] database what is a really good treatment for this type of patient”. “There was discussion about whether or not long-term conditions should include Dementia… We [said] you need to add that as a separate consideration [as it will involve] a completely different kind of tailored support and we want to be able to know what that looks like”.
1b) *Enriched practitioner - patient conversations* “It helps the patient to identify things about their disease and about their health that may not surface otherwise if you don’t ask these questions”. “We ask patients to do the Warwick and then right in front of us we can see a bit about how the patients feeling and lift a conversation out of that and say how does it make you feel to see what you’ve written down”. “It’s very good for building up relationships actually, I know we moan about the paperwork, but sometimes you can get to know different sides to the person. It does make them more open to talking I think sometimes”.	*2b) Empowerment* *encouraging patient engagement in their care through PRMs* “As a diary – print them off put them into a folder… linking to it through their phone”. “[PRM data] can improve their understanding of their disease and what we’re trying to measure … because this is an area that we think is important and should be important to you”. “When they’re reporting and they seem to be getting better, but [their results show] they’re not, you can begin to work with the difference between the two”.	*4b) PRMs enabling ongoing monitoring of patients’ condition and progress with treatment* “So we could have a threshold for a change, for example. So if you, you know, move by two points or one point or whatever, actually that then flags that on to another dashboard that says, this patient…and we can then run a telephone clinic potentially”.
1c) *Creating communication pathways between healthcare services and patients; creating a more person-focused service* “We were able to say, look this is what patients actually want. This is where we’re not scoring so well, that we don’t provide access in that practice. So, they then turned around and said, okay we will fund”.	2c) “*Headway made with personal goals* Let’s focus on a goal instead, let’s focus on you running the marathon each year and let’s see what we can do about that. So, let’s use the PROM and the PREM about this to see how things [get on]”.	4c) *Using PRMs to keep care plans current and relevant* “It was more useful for the clinical changes to drive the frequency of outcome measures. So, we use a measure called Phase of Illness, which captures the context of the current illness; whether somebody is stable, unstable, deteriorating, or dying”.
1d) *Generating pre-consultation communication* “Physicians can see [before the visit] okay, there are some things here that we need to take care of and can identify things about their disease”.		*4d) PRMs enabling remote management of stable patients* “We’ve got cohorts of patients that we know are quite stable; they’re highly educated. They understand what’s required of them. Why do we need to see these patients in clinic or as frequently as we have been? Patients that aren’t terribly engaged, struggle with education and understanding around conditions - surely they’re the ones that we should be concentrating the resources and education on”.

### P3C domain: Information and communication theme 1a) facilitating the presentation of symptom change and current status (practitioner and non-practitioner participant data)

Participants spoke of how it can sometimes be difficult for patients to give specific details on how their symptoms have changed, for example: “I’m not feeling so well right now, but last week I was okay*”,* making it difficult for a practitioner to interpret what change has occurred. This communication barrier can be addressed through PROMs by enabling patients’ subjective feelings about symptom change to be represented by a score. Current scores could be compared to previous scores to give a more accurate depiction of what degree of change had occurred. By posing questions about specific states, PROMs can also help patients to focus on how and what they want to communicate about their current symptoms.

Participants spoke of how different versions of PRMs had been especially designed to help people with difficulties with verbalisation. Participants described their experiences using Talking Mats (a tool designed to help improve the lives of people with communication difficulties by helping them to communicate effectively about things that are important to them) [34] and their own measures (and adjusted scoring schemes) with people who can respond on behalf of patients who are reaching the end of life (proxy measures). The design of alternative versions of standard PRMs may support people who may not have previously been able to voice their own perspective on their health, wellbeing and their experiences of care in becoming more involved in their care.

### Theme 1b) enriched practitioner - patient conversations (practitioner data)

PRMs prompted coverage of issues that may not have otherwise been addressed during the consultation, but which are essential for a holistic outlook on how the patient’s condition is affecting them. Enquiring holistically about a problem i.e. asking about possible psychosocial issues, rather than just biomedical symptoms, may help practitioners to detect emotional aspects to problems that patients may not usually disclose. It may also encourage patient narratives to build during the consultation, by conveying a willingness to deviate from a generic line of practitioner questioning (where the practitioner controls of the direction of the interaction), by instead focusing on what the patient has identified as being as important (through the PRMs); resulting in co-constructed diagnoses and treatment plans [35].

Furthermore, PRMs prompting of holistic dialogue may explain why participants.

reported that PRMs encouraged patient reflection during consultations, as narratives can enhance reflective thinking during medical consultations [36]. Another explanation, provided by one participant, was that reflection was facilitated by patients having to confront their own perspective of their current state of health and/or wellbeing. Though these enriched conversations the practitioner can build a more comprehensive understanding of who the patient is and improve their relationship with the patient.

### Theme1c) creating communication pathways between healthcare services and patients; creating a more person-focused service (practitioner and non-practitioner participant data)

Participants spoke of how professionals’ perspectives on what patients regard as being important, may not match what patients consider most important in relation to their care. PRM data can help make this distinction and justify practitioners’ continued efforts on, and financial support for, areas of care that patients value; enabling practitioners to extend their role as patient advocate. Outcomes based commissioning is another example of how PRMs can be used to enable patients to have a say in what services they receive. This approach to commissioning was being extensively explored by one participant. Lastly, PRMs were creating a communication pathway between patient and services by providing a means for individual practitioner and services to self-appraise; validating cases of good care and affording them the opportunity to improve and become more P3C focused.

### Theme 1d) generating pre-consultation communication (practitioner and non-practitioner participant data)

Three participants stressed the benefits of feedback processes commonly found in feed-forward systems [37]. Participants spoke of how pre-consultation data collection afforded patients and practitioners the time to reflect on aspects of the patient’s condition that the patient had already identified as having a significant impact on their health. This process contrasts sharply with what typically happens in general practice/primary care where the doctor has to take a detailed history of the patient’s general health during what is often a time-constrained consultation. With PRMs reducing the need for practitioners to take detailed histories, practitioners are better placed to work with patients on their priorities, place significance on their perspective, and empower patients to co-construct diagnoses and treatment plans. It is important to note that 2/3 participants that contributed to this sub-theme were not from England, which is indicative of literature which suggests that feedforward processes are far more common in the US and Sweden than in the UK [37].

### P3C domain: My goals/outcomes theme 2a) self-management: PRMs can help practitioners identify issues and areas for improvement (practitioner and-non-practitioner participant data)

Participants, especially healthcare professionals who were involved in care planning work *(secondary dataset),* spoke of how the Patient Activation Measure (PAM) [38], a tool designed to measure the knowledge, skills and confidence a person has in managing their own health and care (their ‘activation’ level) [39], had helped them to identify areas of care that patients either had misunderstandings about, or no knowledge of at all. A common area that patients lacked knowledge on was their medication. Initial responses to questions within the PAM were explored during extended discussions, to gauge whether a patient’s initial response to questions were an accurate representation of their level of understanding. When there were discrepancies, practitioners could work to improve understanding and this resulted in patients feeling happier and more confident about self-management.

Extended conversations sometimes provided further details on why, in situations where there was no knowledge, this was the case. For example, because they trusted their doctor they felt no need to understand the reasons behind prescription decisions. They also created opportunities for health practitioners to make sure patients understood what to do in situations where they may need to act on their own (e.g. what to do when conditions become exacerbated). This in turn encouraged patient reflection on why it is important for them to understand their own health needs and care. Without a suitable level of understanding about why they were receiving specific types of care, patients are arguably unable to act, even if there is desire to self-manage.

### Theme 2b) empowerment: Encouraging patient engagement in their care through PRMs (practitioner participant data)

Participants spoke of how PRM feedback processes can encourage patient engagement in their care. This engagement made patients more receptive to information about their conditions, why certain issues relating to their conditions are of importance to practitioners and why they should be considered important by them too.

One participant had first-hand experience of using PROMs in clinical (mental health) practice. He had worked with men at risk of suicide who were difficult to engage with and would not normally access mental health services. He had found that the Warwick Edinburgh Mental Wellbeing Scale (WEMWBS) [38] notably improved their ability to engage with the service. The purpose of this PRM made sense to them; they could link it to their social functioning and review their progress. As a practitioner, he also found the measure to be helpful, as by just watching how a patient fills it in *“*can give a glimpse into [their] psychological functioning*,”* a point which relates back to the theme discussed in the earlier section; ‘PROMs can help improve practitioner understanding of the patient’s current state’.

There was no mention within the data set of how PRMs had been used to improve or encourage the involvement of carers in the patients’ care planning process, or of how they had been used to identify carers’ own needs and preferences.

### Theme 2c) tracking progress made with personal goals (non-practitioner participant data)

One participant discussed how PRMs are being increasingly promoted as tools to track patients’ progress with personal goals that are meaningful to them, for example, running a race or to dress themselves, as well as the ‘biological’ impact of treatment. In their experience, if PRMs are used in this way the scheduling of their completion needs to be dictated by patient need i.e. when they feel it is necessary to reflect on their advancement with a goal, rather than by pre-imposed time scales.

### P3C domain: Shared decision making (practitioner/researcher participant data)

Only one participant (*primary dataset*) contributed to themes in this domain. In brief, this participant stated that pre-collected PRM data had enabled practitioners to capture information essential to shared decision making during consultations. The measures helped practitioners to get a sense of how ready the patient was, and what their preferences were for further treatment. A colleague of this participant had embedded a PRM that gaged whether shared decision-making occurred in clinical encounters, into the electronic medical record in two different types of practices. Patients completed the measure post-consultation. Practitioners were then, after a specified period, given cumulative feedback on patients’ scores; encouraging reflection on their shared decision-making practices. Practitioners had found this to be a useful process and wanted to continue using the measure.

### P3C domain: Care planning

#### Theme 4a) supporting health practitioners to provide tailored care (practitioner and non-practitioner participant data)

The PAM had enabled practices to identify the most relevant practitioner for individuals, based on patients’ needs and preferences. It could be suggested that matching practitioners to patients in this way can help build a maintainable effective and therapeutic relationship, given adequate resourcing. There were plans to upscale this approach at a wider locality level, so that assignment of professionals from multi-disciplinary teams for chronic conditions to individual patients could be based on activation scores; enabling integration within services to be further developed.

Several initiatives that were in the early stages of being implemented were raised. While the impact of these initiatives on P3C could not be commented on, the intended course of action did describe clear opportunities for P3C enhancement. One was a plan to embed PRM data (“health scores”) into a GP system that a patient’s entire health care team could access. This would enable health professionals, who were based in the community and who had more one-on-one contact with patients, to decide the most appropriate treatment and healthcare goals for individual patients. Continued monitoring of these patients’ PRM scores would then enable the outcome of these decisions to be reviewed and treatment plans to be modified if necessary.

Other participants spoke of the benefits of being able to access PRM databases covering treatment outcomes for population groups when tailoring treatment decisions for individual cases. While the argument that using risk stratification processes to tailor care decisions as an example of P3C practice may be considered tenuous, a more customised example of how PROM based risk stratification can help tailor treatment decisions, which perhaps carries stronger weight was also reported. In this instance, the health service wanted to consider patients’ own unique collection of long-term conditions when creating a tailored plan of care, rather than just offering a set treatment package for people with long-term conditions.

#### Theme 4b) enabling ongoing monitoring of patients’ condition and progress with treatment (practitioner participant data)

Some of the participants had experience of using PRM data to monitor patients’ conditions and progress with treatment. This was done by practitioners independently and by teams of professionals during team meetings. Both resulted in changes to care plans if the PRM data suggested that symptoms were not improving. Being able to see the beneficial impact of treatment plans, through PROM data, was reported as highly rewarding. It was hoped that in the future, efforts to monitor patients though PRMs could be extended through an alert system that would enable salient changes in symptoms (reported through PROMs) to trigger a call from their clinic. In addition, they hoped the alert system could signal negative feedback about experience of care (PREM data), so that practitioners could be made aware of how their service may be failing patients as early as possible.

#### Theme 4c) keeping care plans current and relevant (practitioner/researcher paticipant data)

One participant discussed how it was important to capture the fluid “context of the current illness” in clinical practice, especially for patients nearing the end of life, as phases of illness will change quickly. This participant had captured this information through the ‘Phase of Illness’, measure [40]. By capturing the phase of the illness, practitioners can: determine what the current, best outcome would be for a patient; assess the suitability of current care plans and whether carer’s (changing) needs are continuing to be met; inform the allocation of resources within a team and during the triage process and make referrals to palliative care services (if used outside a palliative care context) timelier [40]. It was stressed that to be able to capture such data, the completion time of the PRM needs to be based on clinical changes, rather than fixed time intervals.

#### Theme 4d) enabling remote management of stable patients (practitioner participant data)

Another participant spoke of how they were increasingly recognising that their patient group had varying needs, requiring different levels of ongoing support. However, they were presently seeing everyone regularly at their clinic, irrespective of need. They proposed that PRMs could be used to identify when, where and how frequently their patients were contacted, and their cases reviewed. This makes contact with patients more appropriate and responsive and resource deployment more cost-effective.

#### P3C domain: Transitions

This domain refers to the care that is required to assist patients whose care is transitioning *across* service boundaries. Continuity of care is required during this period to help maintain regular contact and ensure follow up appointments are made and attended. Participants did not mention any application of PRMs to directly enhance continuity of care *across* healthcare services. However, many of the themes mapped to the domain ‘care planning’ could be viewed as having the potential to improve continuity of care *within* a service. Therefore, if the themes has been mapped to this type of continuity of care the themes may have been mapped slightly differently.

#### Barriers and facilitators to practitioners using PRMs to enhance P3C

A vast array of barriers affecting the successful implementation of PRMs were mentioned by respondents, suggesting that while PRMs can enhance P3C in practice, the realisation of these benefits is contingent on a number of factors, such as whether the practice environment supports their implementation. All practice level barriers are provided in Table 4. Where possible, facilitative actions, mentioned by participants, that can prevent, or limit the impact of the barriers presented, are matched to barriers. The barriers stated can be broadly grouped into 3 categories: people based, questionnaire based and barriers relating to access and interpretation of PRM data. Examples of category 1 (‘people based’) include a lack of training on how to deliver PRMs, practitioners fearing negative feedback and patients mistrusting their purpose. Examples of poor questionnaire design (category 2) include e.g. lengthy questionnaires, inappropriate wording and their inability to measure what it intended to capture. Numbers of barriers and facilitators presented were evenly matched apart for the last category (3): access and interpretation of PRM data. This suggests that while practitioners find implementing PRM problematic, experience and increasing support from the academic community is providing knowledge on how to improve implementation. However, perhaps due to the infancy and lack of standardised feedback processes for PRM use in UK primary care, more guidance and resources are required to make PRM feedback simultaneously accessible and useful to patients, clinical commissioning groups as well as practitioners.

**Table 4:** Barriers and Facilitators affecting practitioners’ ability to use PRMs to improve P3C

	Barrier	Examples	Facilitators
People based	Clinicians’ lack skills for using PRMs	Lack of clarity about the purpose and value of PRMs will fail to motivate patients to complete it and professionals to champion it. Lack of understanding and/or training on how to apply the measure in clinical settings. Requiring the skill to use the measures, whist maintaining rapport with the patient.	Provision of training to practitioners on why PRMs are important e.g., how it fits into P3C theory, how it can be delivered and used in practice to improve service delivery. Showing the patient the findings on the computer screen, while discussing them during consultations.
	Imposed work burden on staff	Staff can view measurement systems as extra and unnecessary work. Health professionals are too overwhelmed by existing workloads, so it would be better if they were not responsible for patients completing measures.	Offering a financial incentive. Using a champion from the same healthcare service to encourage use of the measure. Reducing the burden of the new workflow by training specific staff members to handle the measurement system. Facilitating a smooth integration of the PRM data into a health organisation’s electronic record system, so that accessing it is less burdensome and so that the information integrates with what data is already being collected.
	Emotional burden on staff	Staff resistance to delivering the measures and hearing results, due to a fear of the unknown e.g. what feedback they may receive about their work.	Focusing on the change and improvement that can be made because of the information retrieved from the measure, rather than on what has gone wrong.
	Burden on patients	“Culture shock for patients” – patients are not used to being asked to do ‘homework’ outside of the consultation; being involved in the consultation or being asked new, difficult questions. Patients not motivated to complete the measure as view it as only being useful for the health professional. Completing measures can be time consuming and burdensome. If many measures are given to the patient, they may develop questionnaire fatigue, especially if not thanked or told why the results are important. Technology - if the delivery of the measure becomes electronic then it can introduce a new workflow for the patients (as well as staff). Interface of the electronic version of the measure may not be user friendly.	Making patients aware of improvements to patient care that were made in response PRM data. Ensuring that someone asks the patient to complete the measure, rather than just having it lying around. Monitoring how many questionnaires individual patients are receiving. Picking measures that are relevant to the patient and adding to the collection slowly. Using one measure that can give patients the opportunity to talk about everything, not just certain conditions or issues. Providing different delivery formats, so that the completion of the PRMs is as easy as possible and the offer of support if patients are making a switch to electronic methods. Improving technology, so that patient access is improved. Using external software agencies for IT support and for sharing patient feedback on the website.
PRM based	PRM design	Lengthy questionnaires can interfere with patient-practitioner conversation. It will also make completion even less likely for people who already find it difficult to fill them in. Questions can be hard to understand and/or to respond to for some patient groups due to question design or because of their condition. Translations needed for different languages and for linguistic variations between different English speaking countries. Insensitive question design can have a detrimental impact on the respondent. Family involvement and perspectives not often sought with PRMs.	Working with the developers to make the items are relevant and fitting with your population group. Making decisions on whether people are able to respond to PRMs despite the impact of their condition(s) (e.g. cognitive impairments) on a case-by-case basis. Using proxy measures rather than excluding people who are unable to self-report Triangulating results from patients, health care practitioners, carers and with standard responses from people with the same condition. Using PRMs that use lay language. Using measures that have translated versions available. Making sure that the questions asked are in their local language and are asked by someone that they trust. Using measures that use positively framed items such as the WEMWBS.
	PRMs not providing an accurate measurement of outcome/behaviour/experience	Practitioners find that PAM results often jar with what they have learnt from interacting with patients. Consequently, they doubt whether the measure provides a true representation of how activated someone is. Patients giving answers that they think the practitioner wants or feel nervous about complaining.	Using peer advocates who can advise on how to complete the measures and encourage honest responses. Keeping participants responses anonymous so that respondent bias can be minimised. However, this makes collating data with other sources difficult. Training staff to interpret patient behaviours to combat discrepancies between proxy and self-report measures. Examining variables influencing agreement/disagreement between a proxy and a patient score. Once discrepancies are identified, scores can be adjusted and controlled for.
Access and interpretation	Maintaining patient contact	Can be difficult to feedback to patients who just disappear.	
	Maintaining access to data.	Data given to Clinical Commissioning Groups for aggregate measurements, but data not returned for practice level use.	
	PRMs data difficult to interpret	Findings presented in overly statistical form to people without the skills to interpret them.	Giving a simple overview of the data, showing trends that indicate what might and might not be a good direction to go in. Giving different options for how to make changes in care. Including graphical representations of data and a decision support system. Keeping it simple – limiting the number of questions you use, so you know what good will look like. If the answer options are related to outcomes that are important to patients, the results will be easier to evaluate and will be valued.
	Lack of feedback systems	If a patient accesses their results without an explanation, it can cause confusion and worry.	

## Discussion

### Summary of findings and comparisons with existing literature

The interviewees in this study presented a number of ways in which PRMs directly enhance some aspects of P3C, most typically the interpersonal domains. In the sections below, key findings related to each of the P3C domains are summarised.

### Communication

Themes relating to this domain were prevalent within both data sets, giving weight to previous suggestions that PRMs can enhance communication (e.g. [32]). In addition to collaborating previous assertions, our findings gave a unique insight into *how* communication was enhanced during consultations, something few other studies have attended to [41]. For example, our findings aligned with Greenhalgh et al’s [14] conclusion that PROMs did not significantly change how doctors communicated with patients, instead they enhanced communication by supporting patients to disclose by, as Santana and Feeny [32] hypothesised, helping patients to express their symptoms more succinctly. We also found that PRMs helped practitioners and patients to focus in on what was important to the patient; supported reflective thinking for both practitioners and patients and created an opportunity for holistic questioning. These communication processes support the elicitation of the patient narrative, a style of communication that has been to shown to facilitate a shift away from standardised, passive interactions, associated with the biomedical model, towards person centred dialogue [42]. They are also crucial to relationship building within a consultation.

### Goals/outcomes and care planning

Themes relating to these domains were also recurrent within both datasets and again, collaborated many of Santana and Feeny’s [32] theoretical proposals for how PRMs could enhance P3C. We found that PRMs provided insight into the patient’s perspective and condition. This enabled tailored and reactive care and co-constructed objectives. PRMs identified and addressed errors or gaps in the patient’s knowledge about their condition and care plan. This created opportunities for patients to become more informed on why they needed to engage in their care and to become so. The potential value of such insight has been discussed elsewhere. For example, studies have suggested that collection and consideration of the patient’s point of view could increase treatment adherence and improve patient satisfaction [43] and reduce the number of ‘no-shows’ to medical appointments [44].

Notably, one important and possible use of PRMs not reported by participants, was gaining the carer’s/family’s perspective and engaging them in care planning. During the development of an online compendium of PRMs for P3C, and public participation involvement (PPI) sessions, we established that while existing PRMs do capture carers’ perspectives of how their role as a carer impact them, PRMs rarely, or only briefly try and seek carers’ perspective on the care being received by the person they are caring for. This needs to be addressed for PRMs to utilise carers’ knowledge, perspectives and experiences within patient care planning processes.

### Shared decision making and transitions

These domains were noticeably underrepresented (Shared Decision Making) or not addressed at all (Transitions) in any of the analytic themes, which suggests that, based on the participants’ experiences, PRMs had less impact on these aspects of P3C in practice. This finding could be due to a number of reasons, for example, participants may have had limited knowledge of PRMs that relate to P3C or been instructed to use a selected few; participants may have been more focused on different aspects of P3C or PRMs are less useful to the enhancement of these areas of P3C. Future work would need to unpack these possibilities, bearing in mind the current dearth of PRMs for transitions and how this might be responsible for the absence of evidence surrounding their useful application.

### The importance of a ‘whole service’ approach to PRM application

While individual practitioners can use PRMs for P3C related activities, the success and standardisation of these applications depends on whole service approach to PRM implementation, as PRM use needs to fit into or help re-design how care is currently organised and delivered. Olsen, Aisner and McGinnis [45] advocate a ‘learning health care systems:’ “A system in which data on outcomes are routinely collected, that data are used to identify areas for improvement, new initiatives to address those areas are undertaken, and data on results of change are policy are examined perhaps leading to change in delivery” ([32], pg. 1511). Within our dataset, there were several accounts of how PRMs were being used to create a service that learnt from PRMs at both individual and service-level. Practitioners were using PRMs during multi-disciplinary meetings to discuss progress (or regression) in individual cases and to reflect on the service’s overall management of patient caseload and inform individual practitioner appraisals. PRMs were also being used to bridge the gap between individual patients and service professionals, by giving them an aggregated opinion on what was working well, not so well and where money should be spent; creating an opportunity for both the service and individual consultations to become more person centred.

The barriers raised by participants lend further support to the argument that a whole.

service approach to PRM use is necessary if PRMs are to generate P3C improvements, as many were caused by a lack of a coordinated approach to PRM implementation [3]. For example, patients had perhaps not been involved in decision-making regarding which questionnaires to use and therefore, the wording and design of questionnaires selected sometimes jarred. Staff members were not adequately trained or briefed about the purpose of the measures and were therefore un-motivated and resistant about implementing them and patients were fatigued or confused about the purpose of the measures, as they were not being provided with any feed-back from results or told of how they had informed change.

### Future research and possible application of findings

Whilst thematic studies, such as this one can identify PRM related activities occurring within patient-practitioner consultations that enhance certain components of P3C, further research is needed to unpack how the patient and the practitioner reference and use PRMs during consultations through their talk. Greenhalgh et al. [46] used Conversation Analysis (CA) to explore how PROM data was referred to during oncology consultations. Their main findings were: (1) PROM data was used by practitioners as an independent form of support and justification for their treatment decisions. (2) Explicit reference to PROMs data can create opportunities for patients to disclose other problems (e.g. side effects of chemotherapy). However, practitioners used many communication strategies to curtail discussion of non-cancer related issues. (3) Within their dataset, practitioners typically did not explicitly refer to PROMs data. These findings led the authors to conclude that while PRM can help patients to disclose more, practitioners might not feel equipped to deal with these types of conversations or to know when and how to introduce PRM data during consultations. In other words, while PRMs can enhance practitioner-patient communication, the way in which PRM data is fed back will affect the extent to which this benefit can be realised.

Based on the findings presented here and Greenhalgh et al’s [46] study, it is clear that both CA and thematic analysis would be useful approaches for future studies exploring how PRMs can be used to enhance P3C in other settings and population groups, especially long-term conditions within primary care (a context recognised as being potentially supported by PRM use [46]. Collectively, these methodologies could provide empirical evidence of: 1) how PRMs can be used by practitioners (and patients) to enhance P3C in these specific settings/population groups (thematic analysis), and 2) how specific communication practices by both parties can influence the extent to which these potential P3C applications of PRMs are realised during types of consultations (CA). Such evidence would enable researchers to respond to calls for communication training for practitioners on how to use and discuss PRMs during consultations [47].

### Limitations

The design of the study was decided to an extent by what resources were available to the research team. One consequence of this was that patient perspectives were not collected or commented on. However, patient perspectives have begun to be collected and reported on by the authors [4] and further research will extend this work further, so that practitioner – patient perspectives can be compared in a future study and these findings can be further substantiated. Future studies could also consider using additional qualitative methods to enable data triangulation.

## Conclusion

Practitioners use a vast array of approaches to how to use ***both*** PROMs and PREMs for improving patient care. This resource should be optimised so that professionals can benefit from each other’s learning, overcome barriers to PRM use and work together to extend the potential value that PRMs can offer to P3C delivery. The findings have also help to build a more informed understanding of how P3C components can be translated and enhanced in practice. The study also suggested what P3C domains may currently be best enhanced in healthcare settings through PRM use and which domains are not. This finding provides a rationale for future research to explore whether our findings are replicated in other studies and, if so, what may be causing this lack of domain coverage. For example, whether it’s due to a lack of awareness of what PRMs are currently available, whether the PRMs for these domains do not lend themselves to this type of use or because there needs to be more thought given to how these types of PRMs could be used to improve P3C in practice. There is also the possibility that attending to these P3C domains is more salient in certain types of health care settings. Consequently, data collected from: (1) other types of health care settings/professionals to the settings/practitioners sampled from here or (2) just one type of setting/practitioner that was sampled from in this study, but more extensively, would provide examples of PRM use enhancing these types of domains.

## Abbreviations

GPCC: The Gothenburg University Centre for Person-centred Care
iPRO: Individualised Patient Reported measures
NHS: National Health Service
P3C: Person centred coordinated care
PAM: Patient Activation Measure
PCC: Person Centred Care
PREM: Patient Reported Experience Measure
PRMs: Patient Reported Measures
PROM: Patient Reported Outcome Measure
WEMWBS: Warwick Edinburgh Mental Wellbeing Scale
